# Information Dissemination Analysis of Different Media towards the Application for Disaster Pre-Warning

**DOI:** 10.1371/journal.pone.0098649

**Published:** 2014-05-30

**Authors:** Nan Zhang, Hong Huang, Boni Su, Jinlong Zhao, Bo Zhang

**Affiliations:** Institute of Public Safety Research, Department of Engineering Physics, Tsinghua University, Beijing, China; University of Warwick, United Kingdom

## Abstract

Knowing the information dissemination mechanisms of different media and having an efficient information dissemination plan for disaster pre-warning plays a very important role in reducing losses and ensuring the safety of human beings. In this paper we established models of information dissemination for six typical information media, including short message service (SMS), microblogs, news portals, cell phones, television, and oral communication. Then, the information dissemination capability of each medium concerning individuals of different ages, genders, and residential areas was simulated, and the dissemination characteristics were studied. Finally, radar graphs were used to illustrate comprehensive assessments of the six media; these graphs show directly the information dissemination characteristics of all media. The models and the results are essential for improving the efficiency of information dissemination for the purpose of disaster pre-warning and for formulating emergency plans which help to reduce the possibility of injuries, deaths and other losses in a disaster.

## Introduction

Natural and man-made disasters seriously threaten human life and property. A more reliable and efficient pre-warning information dissemination system could improve public emergency responses, and enable people to evacuate and take protective measures before and during a disaster [1]. In the Indian Coast, for example, more than one hundred people could be saved because a scientist, using his cell phone, managed to warn about an imminent serious tsunami caused by an 8.7 magnitude earthquake [2]. Moreover, victims easily survived in an effective and efficient way if they have more detailed information [3]. Therefore, the research on information dissemination is of great theoretical and practical value. This paper focuses on information dissemination relating to disaster pre-warning; it does not concern itself with research subjects such as economic-geographic development [4].

Information media can be divided into social and traditional media. Social media including short messages, microblogs, and news portals, because of their high impact and coverage ratio made possible by developments in information technology [5]–[6], are becoming increasingly popular and therefore critical tools of information dissemination [7]. For instance, they can enhance the decision-making process since more data is provided than it is the case with traditional media [8]. However, some traditional media, including cell phones, television, and oral communication, also play important roles in information dissemination. In some serious disaster cases, when all electronic networks are paralyzed, traditional media such as oral communication, albeit slower, can still be employed [9].

The different characteristics of each information dissemination medium have been studied in different fields. Sattler found that an effective message could be issued by a credible source and transmitted in a quick and stable way through warning message transmission by cell phone and e-mail [10]. Wei analyzed the optimal combination of television broadcasting sequences which ensured the best information dissemination to television viewers [11]. Whittaker researched information management of emails and found habits of email users in information management [12].

Furthermore, there are some studies that looked at information dissemination media in disasters and emergencies. Odeny found that short message services could improve the attendance at post-operative clinic visits after adult male circumcision for HIV prevention [13]. Zhang established that different information media, including cell phones, television and emails, have different information dissemination characteristics in disaster's pre-warning [14]. Shim used wireless TV to improve disaster management and to provide communications for respondents during a natural or man-made disaster [15]. Katada created a simulation model and built a general-purpose system for the efficient study of the dissemination of information concerning disasters and scenarios of information transmission [16]. Zhang used sound trucks to transmit information in an optimal path in the case of network paralysis caused by a serious disaster [17].

Analysis of recent studies reveals that most works focus on a single medium, but neglect detailed comparisons of different media. However, in actual situations, a single information medium cannot ensure the dissemination of large amounts of information. Therefore, each medium should be analyzed and compared to other media to improve overall efficiency of information dissemination.

In this study, information dissemination models of six information media, including short message service (SMS), microblogs, news portals, cell phones, television, and oral communication were developed and the information dissemination characteristics were studied and compared. The capabilities and mechanisms of these information dissemination media were also studied concerning people of different ages, genders and residential areas. The developed models were applied to the city of Beijing. Based on the simulation and effectiveness analysis of all information media, optimized plans and suggestions were put forward to improve the effectiveness of information dissemination during emergencies. The results of this research are useful in the development of a comprehensive information dissemination system to transmit emergency information in an effective way.

## Method

In this study we use the following evaluation indices to compare the different information dissemination characteristics: total coverage of information reception, the time it takes for half of the population to believe the information, frequency of media usage and time, the degree of trust, total cost, and delay time. Among these indices, the degree of trust and frequency of media usage and time can be directly obtained through questionnaires and the total cost can be obtained from the internet: Taobao (the most famous electronic mall in China; URL: www.taobao.com). Another three indices need to be calculated by the computational simulation based on information dissemination models. The models are established considering special information dissemination characteristics such as dissemination mechanisms and individual preference for different media. Some required parameters in the models such as average forwarding times were also collected by questionnaires which were distributed on site. The simulation allows the calculation of the total coverage of information reception, the time it takes for half of the population to believe the information and the delay time. All the parameters mentioned above are obtained from questionnaires and the internet. Different information dissemination models are described below:

### 1 Basic parameters in information dissemination

#### 1.1 Brief introduction of basic parameters

In the information dissemination process effective information dissemination probability and delay time are two important factors. Effective information dissemination probability expresses the probability of a recipient receiving and believing the information after a message is distributed from an information source. Delay time is the time difference between information reception by media and by recipients.

Effective information dissemination probability and delay time are related to service usage and the degree of trust which reflects the probability that people believe the media. For service usage, media usage frequency, media coverage ratio, and forwarding number are thought to be three important components. To obtain the above data, a questionnaire is considered to be a useful tool.

#### 1.2 Questionnaires

In this study 370 questionnaires were filled out (350 of them were available) in May and June, 2013. In the period of disaster pre-warning, individual differences are very obvious in information acquisition and dissemination. The questionnaires include six multiple-choice questions and twenty-nine fill-in questions. We collected the following data: age, gender, educational background, vocation, media usage number (N_use_) and times per day (T_use_), information forwarding number (n_fw_) and probability (p_fw_), and the degree of trust of each of the six information media. All respondents were between the ages of 10 and 80. About half of questionnaires derived from urban areas while the rest were filled out in rural areas.

The questionnaires allowed a detailed analysis of the degree of trust and service usage for short messages, microblogs, news portals, cell phones, television and oral communication. The data are presented in Table.1

**Table 1:** Comparison of the degree of trust and service condition of six media.

	The degree of trust (%)	Media coverage (%)	Usage frequency (times/day) or (minutes/day)	Average forwarding number (persons)
**SMS**	41.3	97.2	>10 times	11.8
**Microblog**	48.3	66.5	7.5 times	132
**News portal**	57.7	85.0	84.5 min	0
**Cell phone**	43.3	99.0	>10 times	9.7
**Television**	79.0	91.7	99.9 min	0
**Oral**	38.91	100.0		3.9

The degree of trust in an information medium reflects its importance, and is crucial in information dissemination [18]. The questionnaires suggest that television, news portals, and microblogs have the three highest degrees of trust (79.0%, 57.7%, and 48.3% respectively); they are followed by cell phones (43.3%), short messages (41.3%) and oral communication (38.91%). Television and news portals have the highest degrees of trust among the six media because these two media are managed by the government.

Media coverage ratio determines whether the media could be used in information dissemination for pre-warning of a disaster. Oral communication, cell phones, and short messages as the top three (100%, 99%, and 97% respectively) had the top three coverage ratios; reflecting our dependence on these media in our daily lives. Microblogs rank at the bottom of the six media types (66%) which is due to personal preference. The analysis of the data on media coverage ratios reveals that the usage coverage is very high although the degree of trust of some media is low.

The frequency of media usage is an important factor, reflects the popularity of the respective medium, and determines the difficulty of information acquisition. Oral communication is the most frequently used medium in our daily lives. Cell phone and short messaging forms of communication come second as they are used more than 10 times per day. Television and news portals as mass media have lower usage numbers but longer watching times. Microblogs are very popular as well (7.5 usage times per day).

Forwarding number indicates the speed of information dissemination from person to person. Strong forwarding capability could lead to rapid information acquisition [19]. Microblogs had the highest average forwarding number (132); followed by short messaging (11.8), cell phone (9.7) and oral communication (3.9). However, television and news portals are one way media transmitting information from a public organization to a wide audience, and cannot be used for forwarding information person to person.

The detailed results of effective information dissemination probability and delay time are introduced and calculated below.

### 2 Model Establishment

The models of six typical information media including short messages, microblogs, news portals, cell phones, television and oral communication could be divided into three types. The first type was based on person to person without geographical limitation, including short messages and cell phones, the initial stage of which may present an exponential growth in recipients. The second type was based on person to person with geographical limitation such as oral communication where information can be disseminated only within a limited distance. In addition, television and news portals can transmit information from a mass media to person with a logarithmic growth in recipients which were defined as the third type. In order to establish the information dissemination model of the media, we assigned three statuses to the people who are facing the disaster: “ignorance”, “receiver”, and “believer”. “Ignorance” is assigned to people who have not received information about the disaster; “receiver” refers to people who get the information but do not believe it; “believer” designates people who have received the information and believed it.

In this study the information dissemination models of microblogs, oral communication and television were taken as the representative media to analyze. The effective information dissemination probability and delay time of each information medium were obtained through process analysis and data calculation.

#### 2.1 Microblog

**Figure 1:**
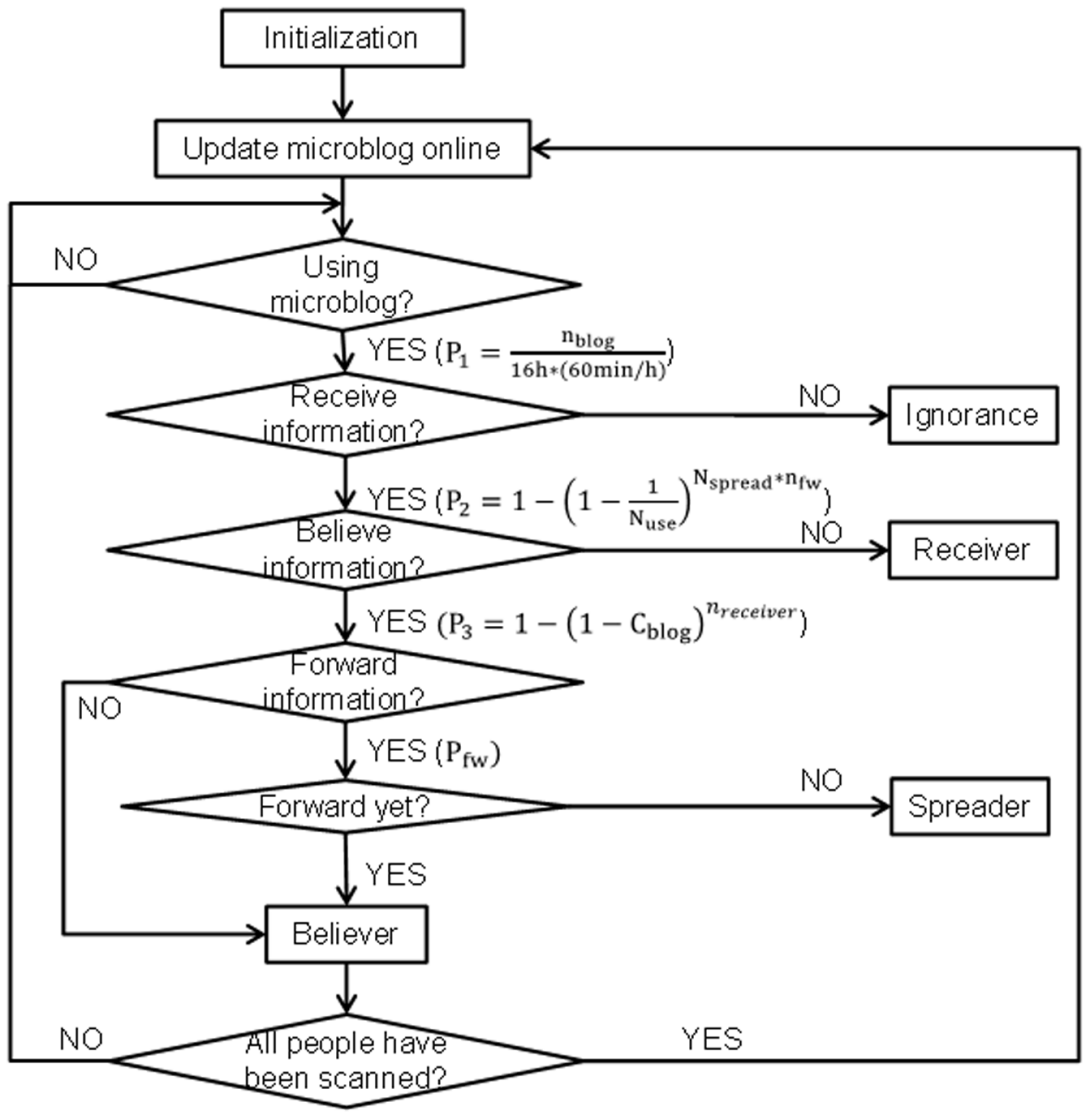
Microblog information dissemination process.

Due to fast speed and convenient operation in information dissemination, microblogs have become increasingly popular in recent years. Fig. 1 shows the information dissemination process of a microblog and the probability of a person being assigned to the status of ignorance, receiver or believer. In Fig. 1 P_1(b)_ expresses the probability of microblog users using a microblog per minute, and n_blog_ is the average usage number of microblog users (16 hours are the effective time per day). P_2(b)_ is the probability of ignorant users receiving information and N_use_ is the number of people using a microblog. N_sp(b)_ is the number of spreaders N_sp(b)_ = N_bel(b)_*P_fw(b)_, N_bel(b)_ is the number of information believers and n_fw(b)_ is the average number of microblog fans (The parameter n_blog_, N_use(b)_, n_fw(b)_ and p_fw(b)_ can be directly obtained from questionnaires). P_3(b)_ is the probability of that those people who received information believe the same; it is related to the degree of trust of the microblog and received information numbers in the time interval (n_rec(b)_ can be obtained by computational simulation). The detailed simulation process is listed below:

1. Set all the parameters which are obtained from questionnaires to target people and create 5 initiative believers which are regarded as the information sources forwarding the information through microblog.
2. Search for the target people who have qualification to forward microblogs in this step. These target people should follow four conditions:
  - The person is using microblog at this step
  - The person is the information believer
  - The person wants to forward the microblog
  - The person hasn't forward the microblog yet
3. Update all the microblog online users.
4. The microblog user i checks the blog.

With the increase of received information number and the degree of trust, the probability will also rise. P_fw(b)_ is the average forwarding probability from believers to spreaders.

Based on Fig. 1, the effective information dissemination probability P_blog_ is expressed by Equation 1. 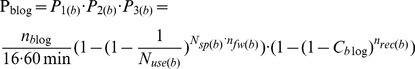
(1)


**Figure 2:**
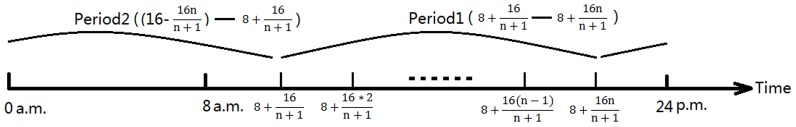
Microblog delay time calculation.

Fig. 2 shows the calculation of delay time of a microblog T_blog_. Users are hypothesized not to check microblogs at night (midnight to 8 a.m.). Based on the usage number of microblogs per day, people check microblogs n times between 8 a.m. to midnight with the same interval 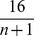. In Fig. 2 period 1 is the checking time and period 2 is the rest time without checking microblogs.

  - Microblog (received information at period 1)

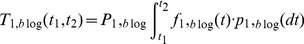
(2)Here T_1,blog_ is the total delay time, P_1,blog_ denotes the proportion of period 1 to 24 hours; f_1,blog_ is the function of delay time; P_1,blog_ represents the time weight of dt.
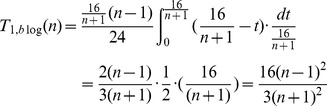
(3)

  - Microblog (received information at period 2)
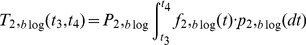
(4)where T_2,blog_ is the total delay time, P_1,blog_ is the proportion of period 2 to 24 hours; f2,blog shows the function of delay time; P_2,blog_(dt) expresses the time weight of dt

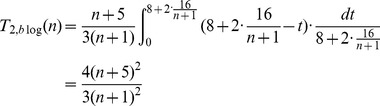
(5)

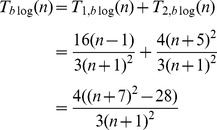
(6)

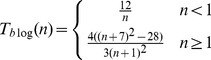
(7)If the usage frequency is less than 1 (n<1), i.e., the microblog user will not check the microblog every day, the average delay time is easy to calculate. Equation 7 is the comprehensive calculation of average delay time of microblog T_blog_ where n is the usage number of the microblogs per day.

Effective information dissemination probability can be obtained through the same analysis since the information dissemination models of short messaging and cell phones are very similar to that of a microblog. The delay time for short messaging was directly obtained through questionnaires. The delay time of cell phones was acquired by conducting 150 calling experiments covering different situations, including answering the phone, busy line, powering off, and hanging up. The models for cell phones and short messages are given in the Appendix A and B of Appendix S1 respectively (information dissemination characteristics curves are shown in Fig. S1 and Fig. S2).

#### 2.2 Oral communication

**Figure 3:**
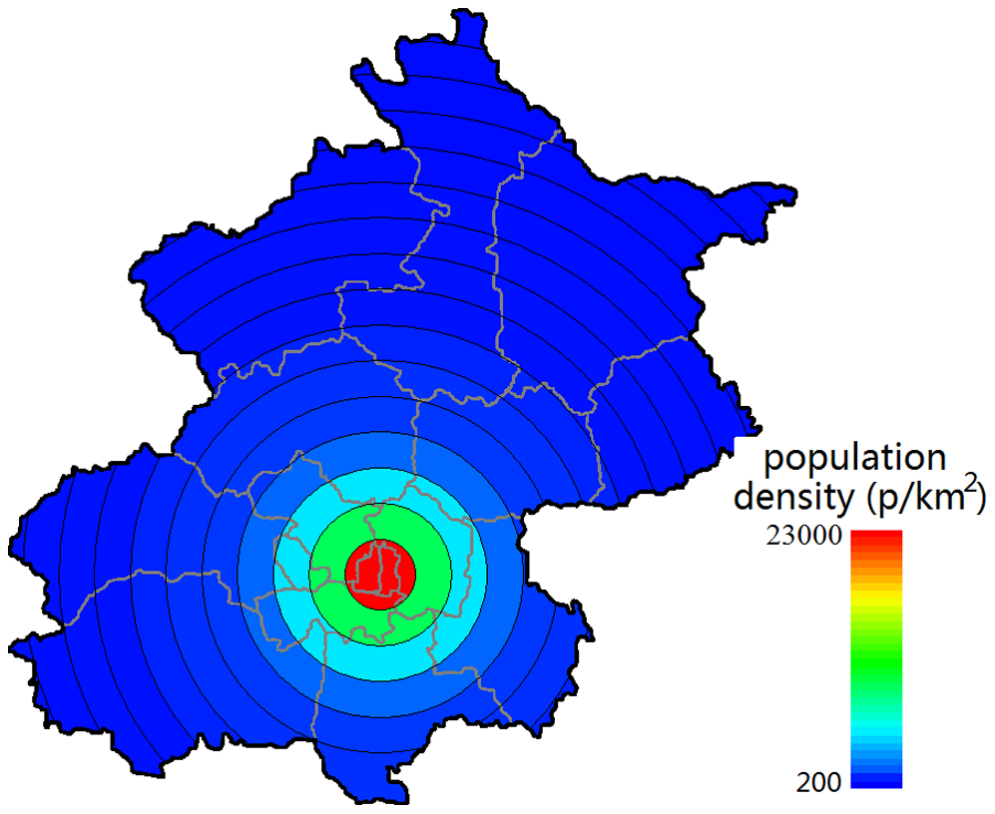
The approximate population distribution map of Beijing.

Oral communication is a very universal and flexible information dissemination form. However, the distance over which information can be transmitted is very limited. Because of this limitation, population density is the most important influencing factor in information dissemination. Beijing's population density decreases from the center to the periphery in concentric circles. Fig. 3 illustrates this population distribution pattern by dividing Beijing into 16 annular regions. In the urban areas depicted by the center annular region, the population density is 23000 persons/km^2^, while the outermost annular region has a population density of just 200 persons/km^2^ (All data were taken from the 2010 Beijing census.). In addition, the speed of information dissemination will decrease as the distance from the central area increases.

An over 100 times difference in population density among different areas leads to an obvious difference in information dissemination speed between urban areas and rural areas. Considering that the positioning of information sources will strongly influence information dissemination, the Monte Carlo method was used to simulate the information dissemination process. It was calculated that the information believers would notify on average 3.87 target people within a range of 90 m. seventy-nine percent of target people would be notified within the distance of 30 m, 14.6% in the range between 30–60 m, and 6.4% within a greater distance [20]. Therefore, the simulation grid was set to be 30 m*30 m and the Beijing area was divided into more than 20 million grids. The dissemination distance and the number of notified people were then obtained. The information dissemination time in one grid was set to one minute, i.e., all people in this grid can obtain within one minute any information disseminated by sources located in the same grid. The effective information dissemination probability, which is the product of probability of information reception, degree of trust, and average delay time, can be obtained through computational calculation.

#### 2.3 Television

**Figure 4:**
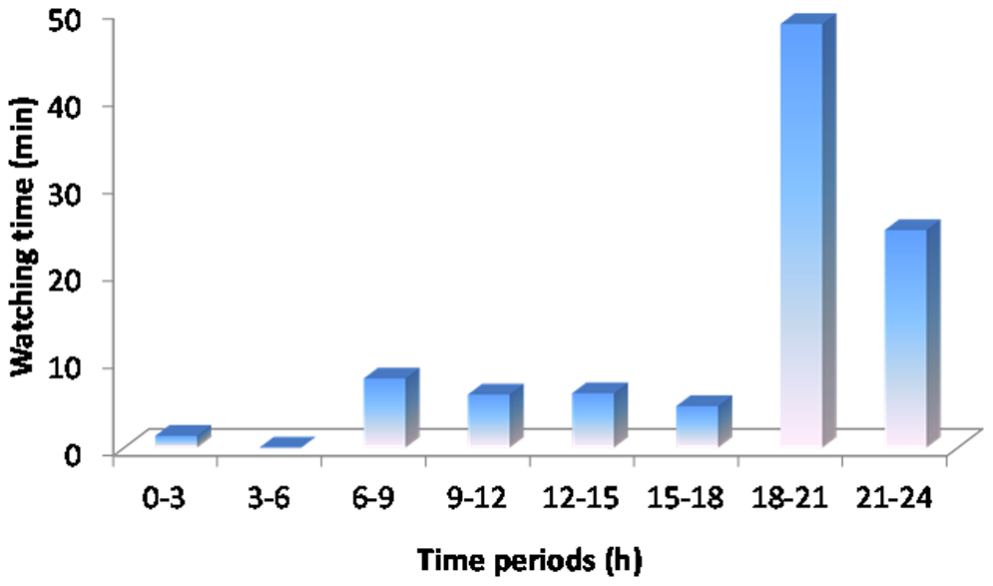
Television watching time distribution.

Television is a mass medium with strong influence and high degree of trust that transmits information from medium to person using images and sound. In this study the television-watching period was divided into 8 phases with 3 hour intervals. Fig. 4 shows the television watching time at different time periods based, on questionnaires. The average time of TV watching peaked at 73 minutes after 6 p.m. which indicates that the majority of people choose to watch TV in the evening. This means that television may be a very good choice for disaster information dissemination during the evening.

**Figure 5:**
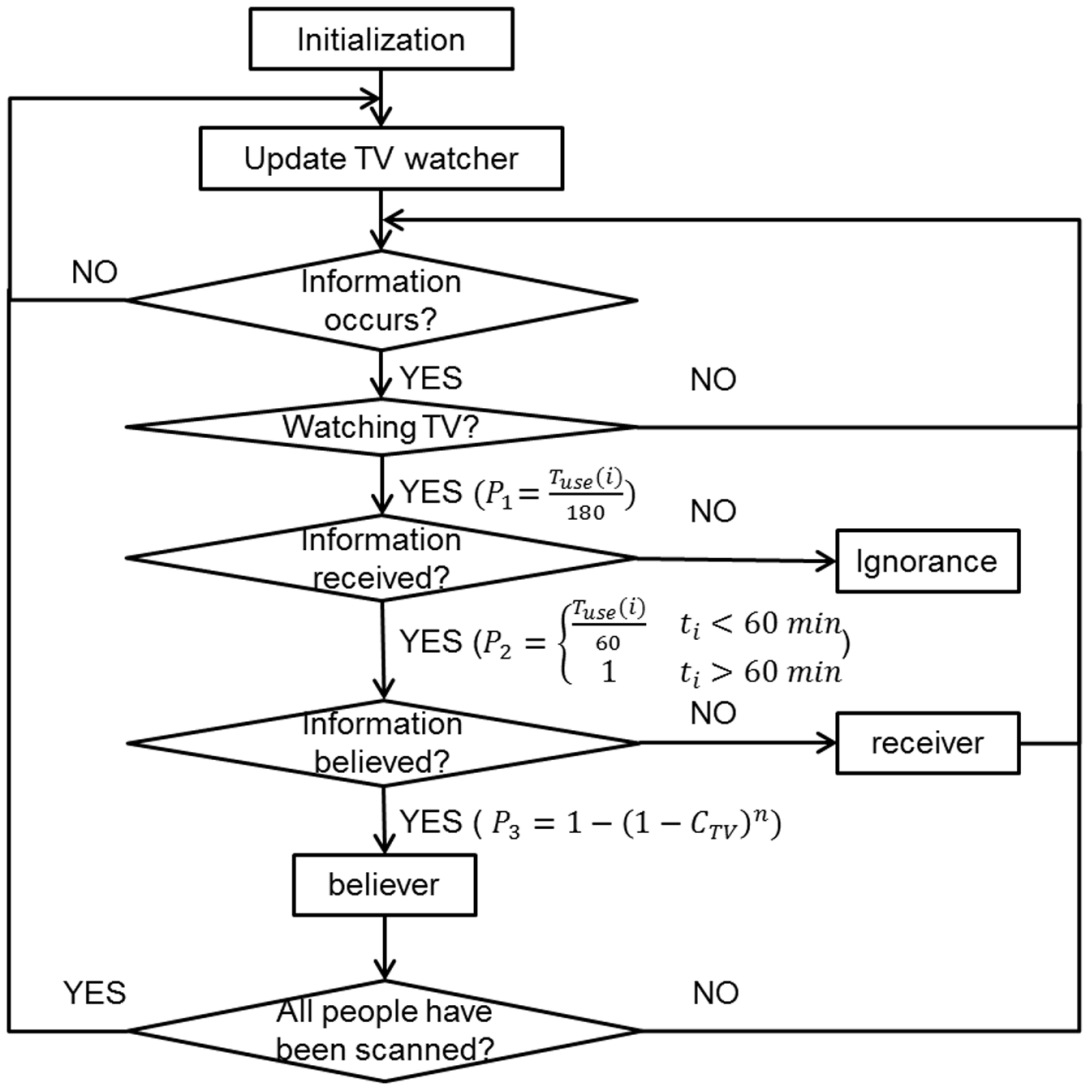
Television information dissemination process.



**Figure 6:**
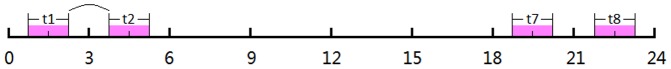
Television delay time calculation.



Fig. 5 shows the information dissemination process of TV. The information is assumed to be broadcast once every hour. Among the processes, P_1,TV_ is the ratio of watching TV in each time period. P_2,TV_ expresses the probability of a TV watcher getting the information from a TV station. A person should get the information at least once during a 60 minute period. P_2,TV_ is therefore a piecewise function related to the duration of watching time t_i_. Finally, the probability that television viewers believe the information P_3,TV_ is calculated through the degree of trust of TV (C_TV_) and receiving times of information n (
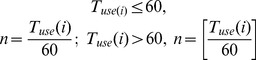
). In summary, the effective information dissemination probability P, is calculated by Equation 8.

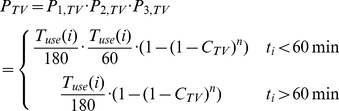
(8)In contrast to a microblog, watching television is a continuous activity. In Fig. 6, the pink color expresses the continuing watching time periods. For example, t1 shows that the watcher watched TV t1 minutes between midnight and 3 a.m. When an information source is broadcast during period 1, the average delay time is calculated based on Equation 9.

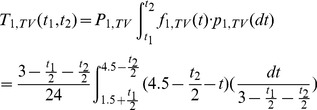
(9)where T_1,TV_ expresses the delay time; P_1,TV_ denotes the proportion of a 24 hour period; f_1,TV_(x) is a function of delay time; P_1,TV_(dt) represents the time weight of dt. The comprehensive function is calculated by Equation 10 considering different delay times from period 1 to period 8.



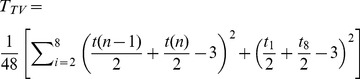
(10)The television model could also be used to simulate the dissemination of information by a news portal because the information dissemination mechanism of a news portal is similar to that of television. The model of a news portal is given in the Appendix C in Appendix S1 (the information dissemination characteristics curve is shown in Fig. S3).

## Results

### 1. Capability analysis of information dissemination

Simulations were performed concerning all the models mentioned above; and different curves were drawn to judge their capability to disseminate information, taking into account typical influencing factors such as age, gender, and residential area. In this study the information dissemination capability is reflected by the number of information believers within a fixed time period. Since the majority of children and elders acquire information from their families, the sample size of this population is small. Thus, we have set the age range from 16 to 55. According to census data [21], there are about 25 million people in Beijing. Using all the data from the simulations, detailed results of information dissemination are analyzed below:

#### Information media 1: short messages

**Figure 7:**
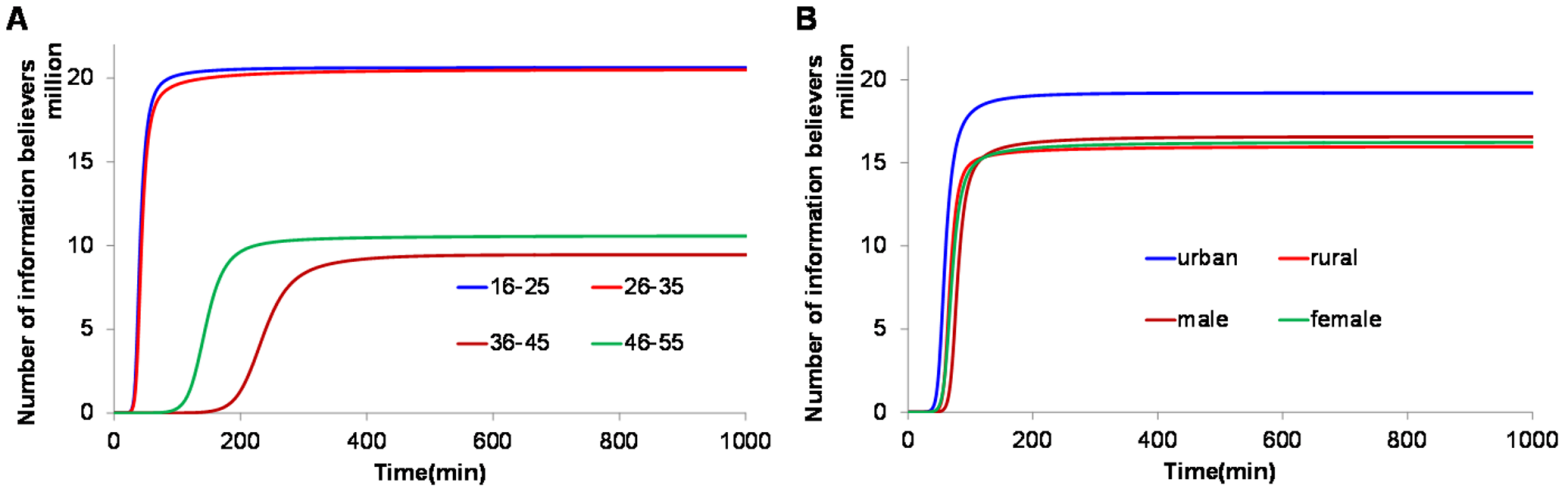
Temporal change of the information believers' number in different groups: short message service. (A) age. (B) gender and residential area.



Fig. 7 shows the information dissemination of short messaging with different influencing factors. The curves indicate that short messages can be sent very rapidly. Statistical data analysis reveals that the information dissemination of SMS accords with equation 11.

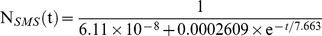
(11)where t is the time for information dissemination and N_SMS_(t) is the total number of information believers. This curve follow the logistic distribution which R^2^ = 0.9963.

Fig. 7 (A) discloses that the capability of disseminating information of the younger group (16–35) is much greater than that of the middle-aged group (36–55). The delay time and usage time are two key factors that explain the difference between various age groups. According to Fig. 7 (B), about 18 million people will receive and believe the information within five hours of information dissemination in the urban area. Fig. 7 (B) also illustrates that the efficiency of information dissemination via short message relies more on the distribution of residential while the impact of gender can be ignored. It can be concluded that increasing short message usage frequency and usage time of people living in rural areas would greatly improve total information dissemination efficiency.

#### Information media 2: microblogs

**Figure 8:**
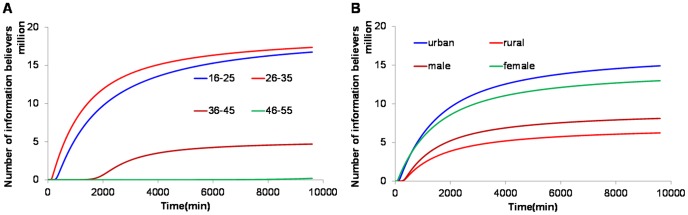
Temporal change of the information believers' number in different groups: microblog. (A) age. (B) gender and residential area.

Fig. 8 demonstrates the information dissemination of a microblog which obeys logarithmic growth. Information source can be transmitted in a short time because there are many microblog fans. Furthermore, the number of people who believe information just reaches about 11 million because of a low degree of trust and low usage. In Fig. 8 (A) we see that the information dissemination effectiveness is low in the group aged 36–55. In contrast, the younger group shows a strong capability in information dissemination. Fig. 8 (B) illustrates that the capability of information dissemination in the urban and female groups is stronger than that in the rural and male group.

#### Information media 3: cell phone

**Figure 9:**
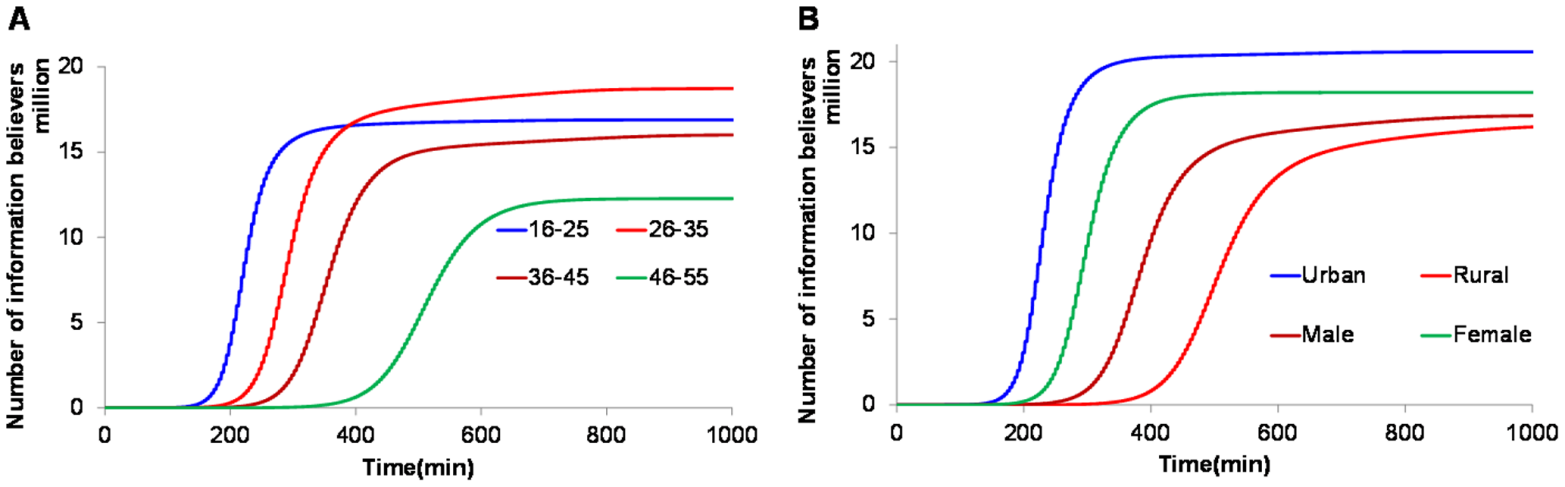
Temporal change of the information believers' number in different groups: cell phone. (A) age. (B) gender and residential area.



Cell phones are the most common information dissemination media used in our daily lives. Fig. 9 reveals that the information dissemination curves of cell phones also fit a logistic distribution with a slow initial dissemination speed. According to our statistical data analysis, the information dissemination of cell phone accords with equation 12.

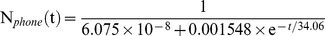
(12)where t is the time for information dissemination and N_phone_(t) is the total number of information believers. This curve follow the logistic distribution which R^2^ = 0.9987.

Fig. 9 shows that more than 10 million people can be informed via cell phone communication within 6 hours after the curves abruptly became flat due to busy lines and powering off of cell phones. Fig. 9 (A) demonstrates that cell phone usage by young people (16–35) is obviously higher than that of middle-aged people (36–55). Fig. 9 (B) shows that after about 16 hours the final value reached 16.8 million, while the rest 8.2 million people had not changed into information believers because of the low degree of trust in cell phones and the usage ratio of rural areas. The unusual continuous increase should be attributed to the long communication time periods via cell phones (about three hours). It also can be seen that females have a higher capability of information dissemination by cell phone than males. In addition, inhabitants in urban areas use cell phones frequently, which means this medium has a better information dissemination capability there than in rural areas immediately before a disaster situation.

#### Information media 4: Oral communication

**Figure 10:**
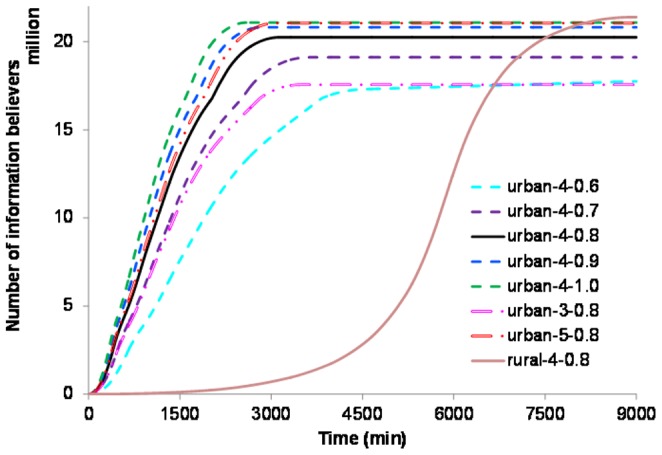
Temporal change of the information believers' number: oral communication.

Fig. 10 shows that an increase in the degree of trust and forwarding number results in an increase of both the growth rate and the final number of information believers. The curves were drawn under different conditions, including different residential areas, forwarding people numbers, and degrees of trust. However, because of the geographical limitation of information dissemination via oral communication, continued increase of forwarding numbers did not result in a significant improvement. A comparison of the black to the brown line shows that, consistent with the law of population density distribution, the speed of information dissemination in urban areas is much higher than in rural areas. Considering that the position of an information source is the main factor determining the speed of information dissemination via oral communication, the Monte Carlo method was employed to improve the accuracy of results and to avoid the uncertainty caused by different information distribution sources. In this case the simulation time is set to 100.

#### Information media 5: Television

**Figure 11:**
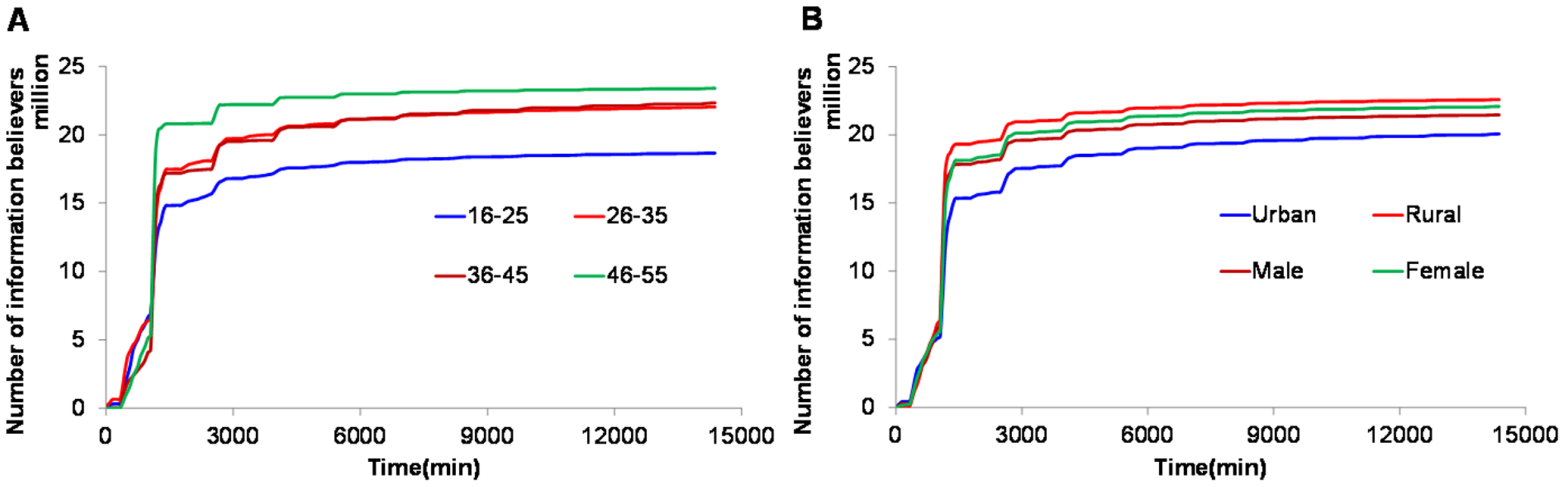
Temporal change of the information believers' number in different groups: television. (A) age. (B) gender and residential area.

Fig. 11 shows the information dissemination by television with different influencing factors. We can conclude that the speed of information dissemination of TV is strongly related to particular time periods: A large number of people are accustomed to watching TV between 6 p.m. and midnight; at other times the speed of information dissemination is more limited. According to the curves of information dissemination, in the first day (1440 min), about 17 million people received the information, and over the following few days the slope of the curve declined. Finally, after ten days, about 22 million people would have been informed via TV because of the high degree of trust. As shown in Fig. 11 (A), the length of time watching TV increases with increasing age, and the speed of information acquisition via TV is faster. Fig. 11 (B) shows that the capability of information dissemination of television in rural areas is, in contrast to all the other five media, stronger than in urban areas. In addition, the effect of gender is very small. Furthermore, the very high information coverage leads to the dominance of TV with regard to information dissemination.

#### Information media 6: News portal

**Figure 12:**
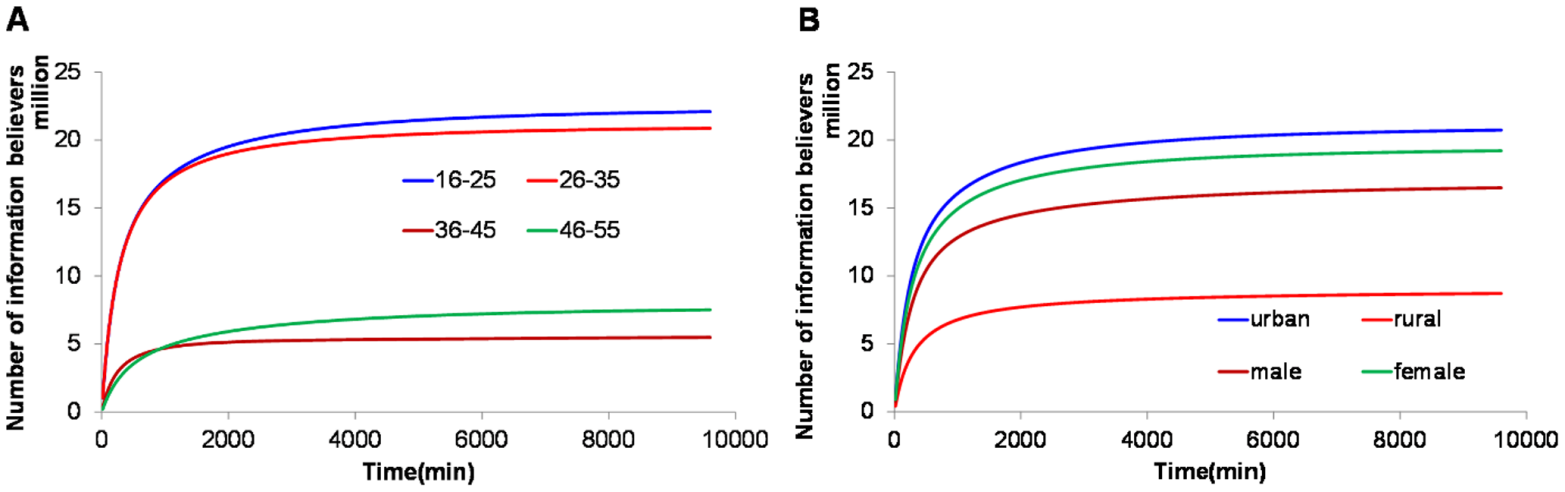
Temporal change of the information believers' number in different groups: news portal. (A) age. (B) gender and residential area.



The information dissemination of a news portal with three influencing factors is shown in Fig. 12. Statistical data analysis shows that the information dissemination of news portal accords with equation 13.

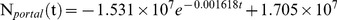
(13)where t is the time for information dissemination and N_portal_(t) is the total number of information believers. This curve follow the logistic distribution which R^2^ = 0.9587.

Fig. 12 (A) demonstrates that age is the most important influencing factor. The fact that information acquisition of the younger group (16–35) is much greater than that of the middle-aged group (36–55) shows that information on news portals can be quickly disseminated among young people. Fig. 12 (B) shows that, considering the influencing factors of gender and residential area, a news portal is very fast at disseminating information, potentially reaching 15 million people in just 1700 minutes. However, due to the lower degree of trust, the final number of information believers reached only 17.8 million.

The analysis of the six media under study increases our understanding of different information characteristics. When a serious disaster is approaching, a single information medium cannot manage the dissemination of a large amount of information. A combination of several information dissemination media which is tailored to the specific situation can increase the efficiency of information dissemination and provide people with more time and more accurate information to be informed and make better decisions. Furthermore, governments can make scientific and correct decisions to transmit information, based on different criteria such as information source and characteristics of disaster carriers.

### 2 Comprehensive assessment of each information dissemination mechanism

**Figure 13:**
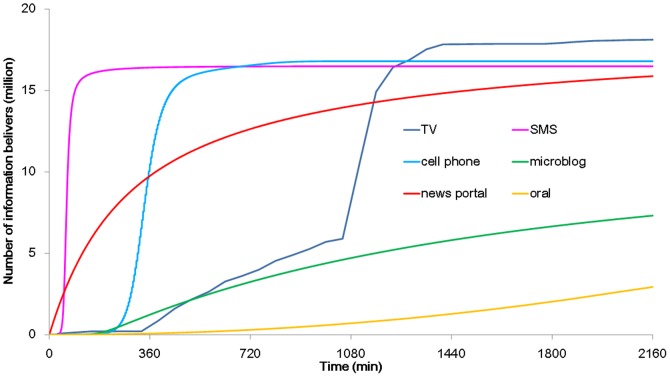
Comprehensive information dissemination comparison of six media.

Fig. 13 shows the change in the number of information believers over time when using short messages, microblogs, cell phones, television, news portals, and oral communication. In the initial 30 minutes, the news portal is the fastest regarding information dissemination because a large number of users can receive the information at the same time. Between 30 and 500 minutes after information dissemination short messages rank first (ignoring the maximum load carrying ability of the base station). The speed of information dissemination can reach exponential growth in the initial period since messages are transmitted from person to person in a short time. After a period of about 100 minutes, the number of information believers will reach a constant value of 16 million. Cell phones have a lower speed of information dissemination than short message services because information cannot be forwarded to many people in a short time. Fig. 13 shows a logistic curve and illustrates that cell phones increased the number of information believers to 16 million within 720 minutes. Television plays an important role in the evening when the majority of people are watching TV at home. With the highest degree of trust and coverage ratio, TV can inform as many people as possible. The information dissemination ability of a microblog is not high due to a lower coverage ratio and degree of trust. It can be used as an auxiliary tool in information dissemination. Oral communication, albeit slow, is a very important information dissemination medium in disaster situations, particularly in the case of network paralysis. A combination of different information media will improve the effectiveness and speed of information dissemination.

In this paper six information media were studied. Six indices were established to evaluate the comprehensive capability of each medium regarding information dissemination: total coverage of information reception (TCIR); the time it takes for half of the population to believe the information (THB); frequency of media usage and time (FMU); the degree of trust (TD); total cost (TC); and delay time (DT).

TCIR is defined as the ratio of the number of people who received the information to the total number of people over a long enough period. THB is the time at which half of the people received and believed the information, and it represents the speed of information dissemination during the initial time. TCIR and THB can be calculated using the model process mentioned above. FMU indicates the popularity of the media. Information from official information media has a high degree of trust. The data for these two indices can be directly obtained from questionnaires. The total cost of each information medium was calculated through a price investigation on Taobao which has more than 70% of electronic commerce market share in China [22]. The delay time of the six information media are calculated by the equations mentioned above.

**Figure 14:**
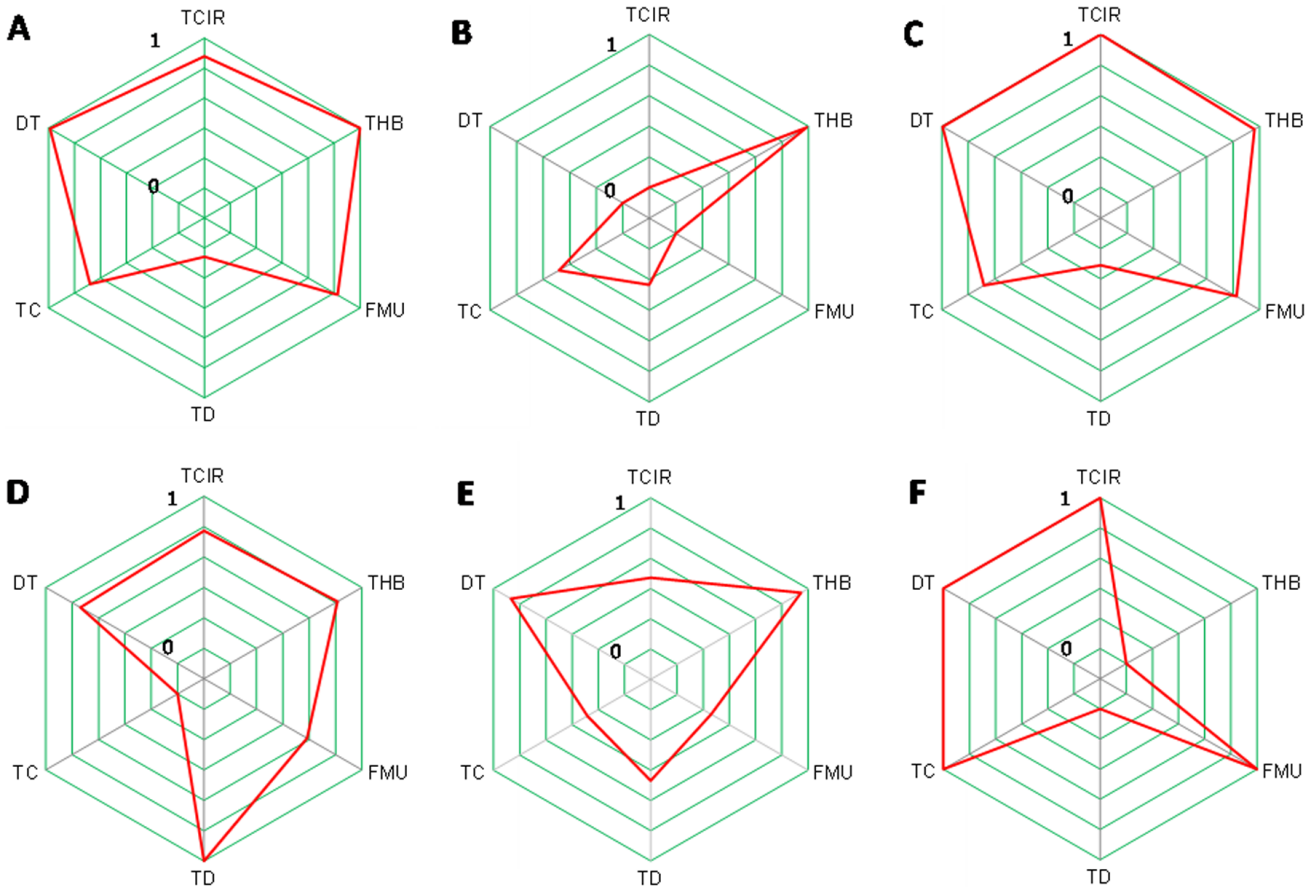
Radar graphs of comprehensive information dissemination assessment of each media. (A) Short message service; (B) Microblog; (C) Cell phone; (D) Television; (E) News portal; (F) Oral communication.

The final scores of the six media are computed using a Min-Max Normalization; they are listed in Table.2. Six indices are classified into two categories. One is positive (+) (capability of information dissemination increases when the value of the index increases), the other is negative (−) (the value is subtracted by 1). According to these six indices, the comprehensive expression of each information medium is reflected in the radar graph shown in Fig. 14.

**Table 2:** Value of six indices of different information media.

		SMS	Microblog	Cell phone	Television	News portal	Oral
**TCIR**	(+)	0.879	0	1.000	0.773	0.472	1.000
**THB**	(−)	1.000	0.986	0.961	0.814	0.947	0
**FMU**	(+)	0.826	0	0.826	0.583	0.261	1.000
**TD**	(+)	0.059	0.235	0.110	1.000	0.468	0
**TC**	(−)	0.682	0.482	0.681	0	0.283	1.000
**DT**	(−)	0.990	0	0.994	0.737	0.867	1.000

The radar graphs express the information dissemination capability of the six media and the comprehensive characteristics directly. Data analysis reveals that short messages, cell phones, and TV have a higher comprehensive information dissemination capability. Short messages and cell phones have a shorter delay time and higher information coverage ratio as well as information dissemination speed, but the degree of trust in them is lower. Television has the highest degree of trust but longer delay time. Microblogs, which have a very long delay time and moderate degrees of trust and information coverage ratio, have a fast initial information dissemination speed. News portals, as a very popular network media, are a very fast method of information dissemination, particularly during the early periods. Oral communication is also a very important information dissemination medium (no cost, ease of use), especially in high population density areas. Different lengths of pre-warning times allow different choices of information dissemination media. For instance, if the pre-warning time is limited, short messages and news portals should be used. Ultimately, the combination of different media can improve the efficiency of information dissemination. The results above can be useful in making an emergency plan that ensures the safety of lives and properties during a disaster.

### 3 Information media in disaster pre-warning

Developing information dissemination technology and understanding the mechanisms of each information medium are crucial for disaster pre-warning and management. Tailored to the specific disaster situation, government and victims can use different information dissemination strategies. Cell phones, SMS, microblogs, news portals, and TV usually play the most important role in some conventional disaster pre-warning such as rainstorm and frost because they feature a higher information dissemination effectiveness. However, when a serious earthquake strikes, the majority of networking and station bases will paralyze and the majority of electronic network media cannot be used. Here, oral communication will disseminate emergency information with few words or sentences. A large number of victims would try to notify all the people around them. Our analysis of oral communication mentioned above suggests that information sources positioning strongly determined the effectiveness of information dissemination. Governments can set the optimal source position to improve the information dissemination speed; they also can assess the spreading time through the analysis introduced above to help improve disaster management and save more lives. Data analysis also revealed that TV has a higher degree of trust and that it also has a higher information dissemination effectiveness in the evening. Governments should put their attention to TV rather than microblog or news portal to spread the warning information if a serious disaster occurs in the evening. Generally speaking, by combining different information media characteristics governments can improve disaster pre-warning and reduce casualties and damage to properties in an effective way, and victims can acquire more information to make informed decisions. Summing up, there is a need to analyze information dissemination characteristics of different media to ensure that warning information can be spread to every person with short delay times in a reliable manner.

## Conclusions

In this study models of six information dissemination media, including short messages, microblogs, cell phones, television, news portals, and oral communication, were established. The capabilities of each medium to disseminate information were assessed, using data obtained from the dissemination models, statistical data, and questionnaires collected in Beijing. Based on the information dissemination capability analysis and taking into consideration factors such as age, gender, and residential area, different characteristics of the six media were summarized. Our analysis shows that SMSs have the highest speed while cell phones can disseminate more detailed information because verbal communication allows better explanation of complex situations. People's habits suggest the employment of television be emphasized in the evening.

In case of serious disasters such as earthquakes, electronic networks are prone to paralyze and oral communication will play an important role to disseminate information in a reliable manner. To directly compare and analyze different aspects of the information dissemination capabilities of the six media radar graphs considering six indices were drawn. Short message services and cell phones have more comprehensive information dissemination capabilities than other information media but they have lower degrees of trust. Television is also a good information dissemination medium; it has a higher information coverage and the highest degree of trust. Compared to other information media, oral communication is not outstanding in information dissemination speed; however, it possesses convenience. News portals and microblogs can be used as auxiliary tools, but their information coverage is not very large. Each of the six media has different strength and limitations; therefore, to help improve dissemination of information, reduce losses, and ensure the safety of disaster carriers, their combination should be tailored to the specific disaster situation. The models and simulation methods can be applied to many other regions.

In future works, more ways of information dissemination and more influencing factors will be considered, including the maximum information carrying capacity and the vulnerability of each medium in a disaster. An integrated system covering all recommendable combinations of media will be established to disseminate emergency information timely and accurately under various circumstances.

## Supporting Information

Appendix S1 (DOCX)

Figure S1 **Cell phone information dissemination process.**
(TIF)

Figure S2 **Short message service information dissemination process.**
(TIF)

Figure S3 **News portal information dissemination process.**
(TIF)
