# Supplementary material for: Information Dissemination Analysis of Different Media towards the Application for Disaster Pre-Warning
Source: PLoS One. 2014 May 30;9(5):e98649. doi: 10.1371/journal.pone.0098649 (PMC4039515; doi:10.1371/journal.pone.0098649)
Supplement: Appendix S1 — (DOCX) [file pone.0098649.s001.docx]

**APPENDIX S1**

**Appendix A. Information dissemination model of cell phone**

Fig. S1 shows the information dissemination process of cell phone. P_1,phone_ is the probability that person’s cell phone receiving the information and it is related to notified people (n’) in current step and total population number N_p_. P_2,phone_ reflects whether people could answer the cell phone and get the information (from the n times cell phone experiments, there are $n_{ans(p)}$ people answer the cell phone). P_3_, which is related to the degree of trust of cell phone ($C_{\mathrm{phone}}$) and received phone numbers ($n_{rec(p)}$ can be obtained by computational simulation) in the time step, is the probability of information relievers to believers. P_4,phone_ is felt to be the probability that people will spread information and the number of spread people ($N_{spr(p)})$ is obtained from questionnaires.

From the process shown in Fig. S1, effective information dissemination probability P_phone_ is expressed in equation 11.

 (11)

Delay time of the cell phone is calculated based on the data of 150 times cell phone experiments. It is a weighted average of delay times in different states including answering phone, busy line, powering off and hanging up.

**Appendix B. Information dissemination model of short message service**

Fig. S2 shows the information dissemination process of short message service. Among that, P_1,SMS_ is the probability of person’s cell phone receiving the message in one step and it is related to number of spreaders (N_spr(SMS)_), average spread number (n_spr(SMS)_) of spreaders and total population number (N_SMS_). P_2,SMS_ is the probability which is related to delay time (t_d (SMS)_) and the time of cell phone received the message (t_rec(SMS)_). P_3,SMS_, which is related to degree of trust of short message ($C_{\mathrm{SMS}}$) and received message number ($n_{rec(SMS)}$) in the time step, is the probability of information relievers to believers. Among the all parameters, N_spr(SMS)_, P_2,SMS_ and n_rec(SMS)_ were obtained by simulation and n_spr(SMS)_, N_SMS_ and C_SMS_ a

From the process shown in Fig. S2, effective information dissemination probability P_SMS_ is expressed in equation 12.

| $P_{\mathrm{SMS}}=P_{1,SMS}*P_{2,SMS}*P_{3,SMS}=\left( 1-\left( 1-\frac{1}{N_{\mathrm{SMS}}} \right)^{N_{spr(SMS)}*n_{spr(SMS)}} \right)\cdot P_{2,SMS}\cdot（1-\left( 1-C_{\mathrm{SMS}} \right)^{n_{rec(SMS)}}）$ | (12) |
| --- | --- |

Average delay time of short message is calculated based on the data of questionnaires.

**Appendix C. Information dissemination model of news portal**

Fig. S3 shows the information dissemination process of news portal. Here, the information is assumed to be broadcasted per half an hour. Among the process, P_1,np_ is the ratio of visiting news portal in the time periods. $P_{2,np}$ expresses the probability of a news portal visitor get the information from website. Believing probability $P_{3,np}$ is calculated through the degree of trust of news portal ($C_{\mathrm{portal}}$) and receiving times of information n ($n=\frac{T_{\mathrm{use}}\left( i \right)}{30}$). In summary, effective information dissemination probability P_np_ is calculated by equation 13.

|  | (13) |
| --- | --- |

Delay time of information dissemination in news portal is similar with television, and the final value is calculated below:

(14)

Where $T_{1,np}$ is total delay time of news portal; $P_{1,np}$ denotes the proportion of each period to 24 hours; $f_{1,np}(t)$ is the function of delay time; $p_{1,np}(dt)$ represents the time weight of dt.

 (15)
