# Supplementary figures and images for: Information Dissemination Analysis of Different Media towards the Application for Disaster Pre-Warning
Source: PLoS One. 2014 May 30;9(5):e98649. doi: 10.1371/journal.pone.0098649 (PMC4039515; doi:10.1371/journal.pone.0098649)

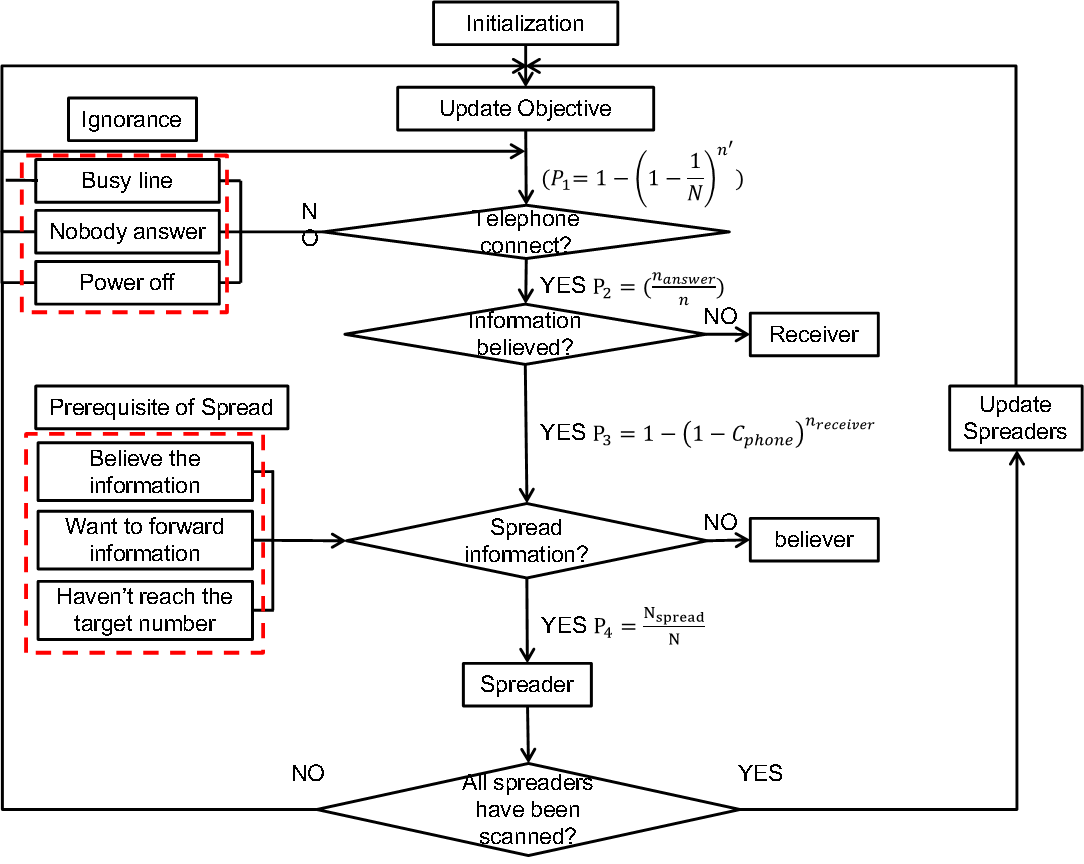

Supplement: Figure S1 — Cell phone information dissemination process. (TIF) [file pone.0098649.s002.tif]

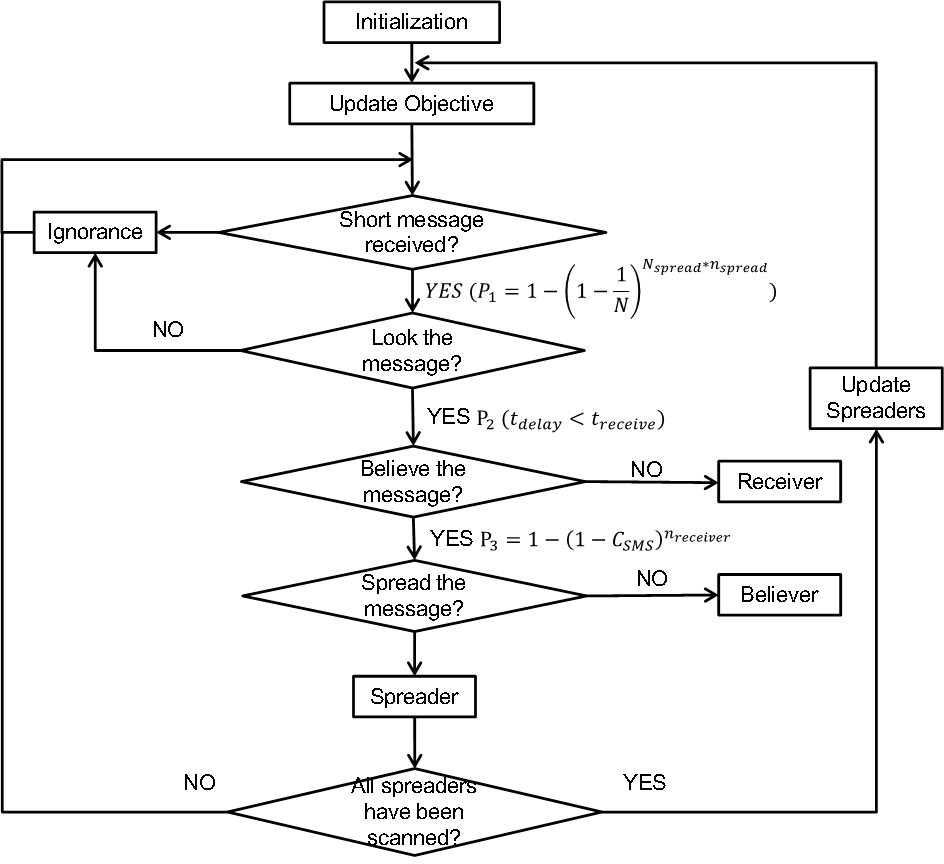

Supplement: Figure S2 — Short message service information dissemination process. (TIF) [file pone.0098649.s003.tif]

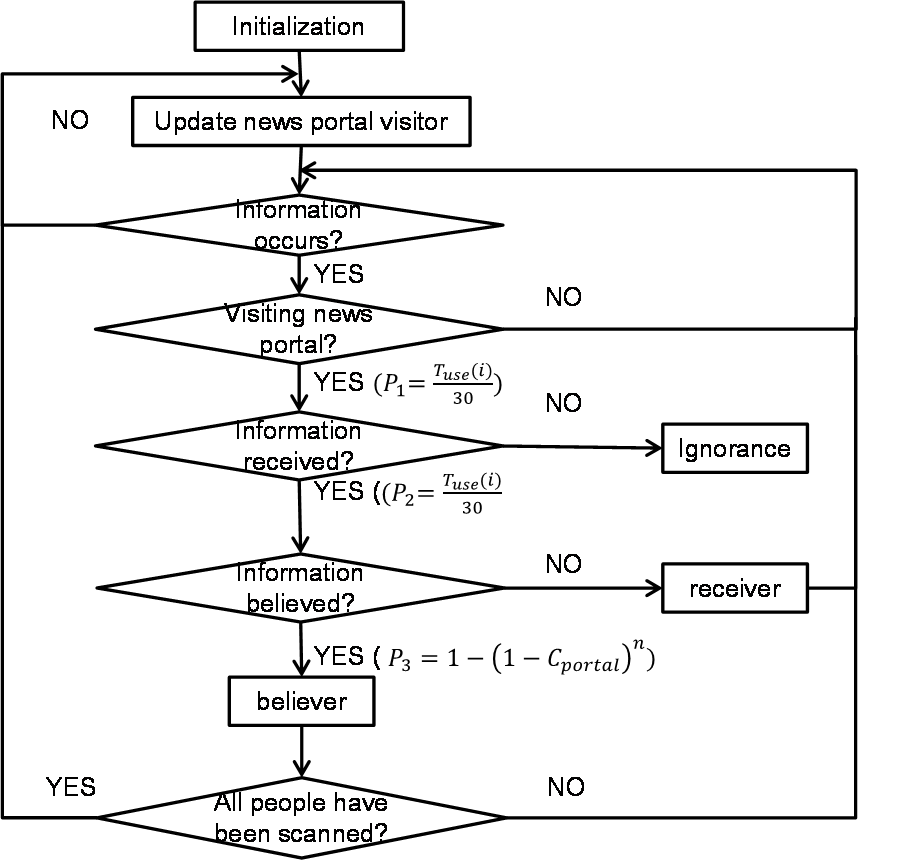

Supplement: Figure S3 — News portal information dissemination process. (TIF) [file pone.0098649.s004.tif]
